# Nonlinear Dynamic Measures of Walking in Healthy Older Adults: A Systematic Scoping Review

**DOI:** 10.3390/s22124408

**Published:** 2022-06-10

**Authors:** Arezoo Amirpourabasi, Sallie E. Lamb, Jia Yi Chow, Geneviève K. R. Williams

**Affiliations:** 1 Sport and Health Sciences Department, College of Life and Environmental Sciences, St Luke’s Campus, University of Exeter, Exeter EX1 2LU, UK; g.k.r.williams@exeter.ac.uk; 2 College of Medicine and Health, St Luke’s Campus, University of Exeter, Exeter EX1 2LU, UK; s.e.lamb@exeter.ac.uk; 3 Physical Education and Sports Science Department, National Institute of Education, Nanyang Technological University, Singapore 637616, Singapore; jiayi.chow@nie.edu.sg

**Keywords:** dynamic stability, Lyapunov exponent, fall risk, walking, biomechanics, nonlinear dynamic analysis, ageing

## Abstract

Background: Maintaining a healthy gait into old age is key to preserving the quality of life and reducing the risk of falling. Nonlinear dynamic analyses (NDAs) are a promising method of identifying characteristics of people who are at risk of falling based on their movement patterns. However, there is a range of NDA measures reported in the literature. The aim of this review was to summarise the variety, characteristics and range of the nonlinear dynamic measurements used to distinguish the gait kinematics of healthy older adults and older adults at risk of falling. Methods: Medline Ovid and Web of Science databases were searched. Forty-six papers were included for full-text review. Data extracted included participant and study design characteristics, fall risk assessment tools, analytical protocols and key results. Results: Among all nonlinear dynamic measures, Lyapunov Exponent (LyE) was most common, followed by entropy and then Fouquet Multipliers (FMs) measures. LyE and Multiscale Entropy (MSE) measures distinguished between older and younger adults and fall-prone versus non-fall-prone older adults. FMs were a less sensitive measure for studying changes in older adults’ gait. Methodology and data analysis procedures for estimating nonlinear dynamic measures differed greatly between studies and are a potential source of variability in cross-study comparisons and in generating reference values. Conclusion: Future studies should develop a standard procedure to apply and estimate LyE and entropy to quantify gait characteristics. This will enable the development of reference values in estimating the risk of falling.

## 1. Introduction

Maintaining a healthy gait into old age is key to preserving the quality of life and reducing the risk of falling. An estimated 28–35% of people older than 65 fall annually [1]. Fractures and serious injuries occur in about one-quarter of falls [1,2]. Age is an independent risk factor for falling, and hence, all older people are at risk of falls. There are four main categories that confer a higher risk of falling: environmental, behavioural, socioeconomic and biological factors [1,3,4]. Biological factors of gait and balance are the most common and important risk factors since they underpin the risks posed by other factors [5]. The chance and extent of gait instability leading to falls also increase with age [6,7,8,9].

There are more than 12 clinical fall risk assessment tools for older people, among which the Berg Balance Scale [10], Functional Reach Test [11], and Timed ‘Up & Go’ test [11], physical and fall self-confidence assessment methods are commonly used for fall prediction. Although clinical assessment could provide useful motor outcomes to detect the early signs of balance alteration and then provide key information about fall risk, they may not be sufficient to predict falls in older populations. For a better understanding of gait instability in older adults and their risk of falling, several biomechanical methods have been developed (for a review, see [12]), with the nonlinear dynamic analysis gaining increasing interest in recent years.

Biomechanical laboratory-based studies have identified characteristics associated with ageing gait and increased fall risk which provide the field with the tools to understand changes in gait characteristics. For example, lower leg strength, gait symmetry proprioception and increasing variability in step width, step length, stride length, step time, stance time, stride velocity and single support are associated with fall risk [12,13,14].

While variability measures feature in traditional linear tools, it is assumed that variability between strides associated with fall risk is random and independent of past and future strides [15]. In contrast, the nonlinear dynamic analysis showed that variations in the time series of specific gait parameters are not random but demonstrate a deterministic behaviour. This behaviour may be partially sensitive to physiological ageing [15,16,17]. Nonlinear dynamic analysis methods provide an opportunity to study the inner structure, regularity, complexity and stability of variables describing gait and have presented strong evidence of being associated with the risk of falling [9,12,17,18,19]. However, there is a range of nonlinear dynamic measures that can be used and a range of ways of calculating each of these measures [12,18].

A question remains as to which nonlinear dynamics techniques are most sensitive and robust in indicating fall risk. While previous systematic reviews in the area have focused on one or a few nonlinear techniques, such as Lyapunov Exponent (LyE) and Floquet Multipliers (FMs) (Bruijn, Meijer [12] and Hamacher, Singh [17]), FMs Riva, Bisi [19] LyE (Mehdizadeh [18]) and entropy (Yentes and Raffalt [20]), there has been no comprehensive review of all nonlinear methods used in studying instability in the gait of older adults.

Therefore, the aim of this review was to summarise research on instability in healthy older adults with and with our history of falling in terms of the (1) nonlinear dynamic measures used, the variety of their calculations and values and (2) methodological issues including age range, walking modality, kinematic variables and clinical risk of falling assessment used. The current study was intended to help future researchers consider the most powerful nonlinear dynamics measures for identifying fall risk and associated experimental designs and calculation methods. Secondly, to capture the current range of values reported for different nonlinear dynamic measures associated with gait and risk of falling in healthy elderly.

## 2. Methods

### 2.1. Search Strategy

An electronic search was performed by one reviewer (AA) in February 2022 to identify papers that quantified nonlinear dynamic characteristics of walking in healthy older adults. The databases searched were Medline Ovid and Web of Science. Reference lists of articles included were checked to make sure that all related papers were considered. The following search terms were used, and both original research and short communications were included:

1. gait.ti,ab.;
2. walk*. ti, ab.;
3. exp Gait Analysis/ or Gait/;
4. 1 or 2 or 3;
5. (dynam* adj2 stabil*). ti, ab.;
6. Lyapunov.ti, ab.;
7. (nonlinear adj2 dynamic*).ti, ab.;
8. 5 or 6 or 7;
9. old*.ti,ab.;
10. elder*.ti, ab.;
11. 9 or 10;
12. 4 and 8 and 11.

### 2.2. Inclusion and Exclusion Criteria

**Figure 1:**
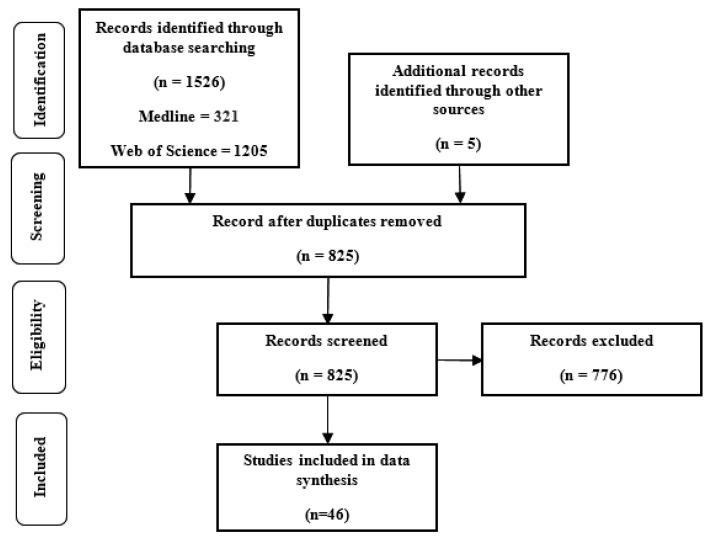
Included research flow diagram based on PRISMA guideline [21].

Two reviewers (AA and GW) assessed the titles and abstracts of the articles. The inclusion criteria were studies of (1) nonlinear dynamic measures of gait or during continuous human walking; (2) English language papers (3) falling or ageing in healthy individuals. The exclusion criteria were studies of (1) humanoid robots; (2) specific diseases or pathology; (3) movements other than walking; (4) conference papers; (5) modelling or simulation studies; (6) animal studies; (7) review or perspective papers. All the papers were quality-assessed using the PRISMA guideline [21]. Figure 1 presents the research flow diagram.

### 2.3. Data Extraction

A customised spreadsheet was used to extract data from the papers associated with the following themes: the population of healthy younger adults and healthy older adults (YO comparison) and older fallers or non-fallers (F-NF); walking modality on the treadmill (TM) or overground (OG); data collection modality divided into four categories: Inertial sensors or accelerometers, goniometers, force plate, and motion capture using passive marker-based and video systems; methods of performing fall risk assessment; nonlinear dynamic analysis employed and methods of calculation used.

### 2.4. Data Visualisation

Reported LyE values were classified based on younger, older, walking conditions (TM walking or OG walking), LyE estimation algorithms (Rosenstein, Wolf, Kantz and Ihlen [22,23,24,25]), data type (velocity, acceleration and joint angle) and direction (anterior-posterior (AP), vertical (VT) and medio-lateral (ML)) to facilitate comparison across studies. In order to visualise the comparison of reported LyE values across studies and their estimated central tendency, boxplots were used. The mean and standard deviation of values for studies were illustrated as point plots and error bars. The points were displayed by a colour to differentiate between different LyE estimation methods and walking conditions. When data were presented only in graphical form in a study, values were extracted by digitising the graphs using PlotDigitizer software (version 4.3., https://apps.automeris.io/wpd/, last accessed date: 22 March 2022). Where multiple gait speeds were reported in a study, the data relating to the speed closest to the preferred walking speed was selected.

### 2.5. Effect Sizes

Cohen’s d was used to quantify the standardised effect sizes in nonlinear dynamic measures between younger adults and older adults (YO) and fall-prone older adults and non-faller older adults (F-NF) groups. Cohen’s d was evaluated using Equations the following equations [26]:

(1) $$\mathrm{Cohen}’s\;d=\left(M_{2}-M_{1}\right)/{\mathrm{SD}}_{\mathrm{pooled}}$$

(2) $${\mathrm{SD}}_{\mathrm{pooled}}=\sqrt{\left(({\mathrm{SD}}_{2}^{2}+{\mathrm{SD}}_{1}^{2})/2\right)}$$

where M_1_ is the mean of the one group of study (i.e., younger); M_2_ is the mean of the other group of study (i.e., older); SD_1_ is the standard deviation of the younger group; SD_2_ is the standard deviation of the older group. Cohen categorised effect sizes, d, into categories, with d < 0.2 as small, 0.2 < d < 0.8 as medium and values greater than 0.8 as large [26].

## 3. Results

The search yielded a total of 1040 articles (Figure 1). After screening, 46 articles were considered eligible for inclusion in this review. Eighteen studies considered research questions focused on the faller–non-faller (F-NF) comparison [27,28,29,30,31,32,33,34,35,36,37,38,39]. Fifteen studies investigated the young–old (YO) comparison [3,6,7,9,38,40,41,42,43,44,45,46,47,48,49]. Six studies conducted F-NF and YO comparisons [33,50,51,52,53,54]. Nine papers studied older adults only [30,31,55,56,57,58,59,60,61]. Three studies compared LyE values for the same population in different conditions (OG or TM walking) [6,29,59] or different LyE estimation methods [29].

### 3.1. Study Design Characteristics

#### 3.1.1. Population

**Figure 2:**
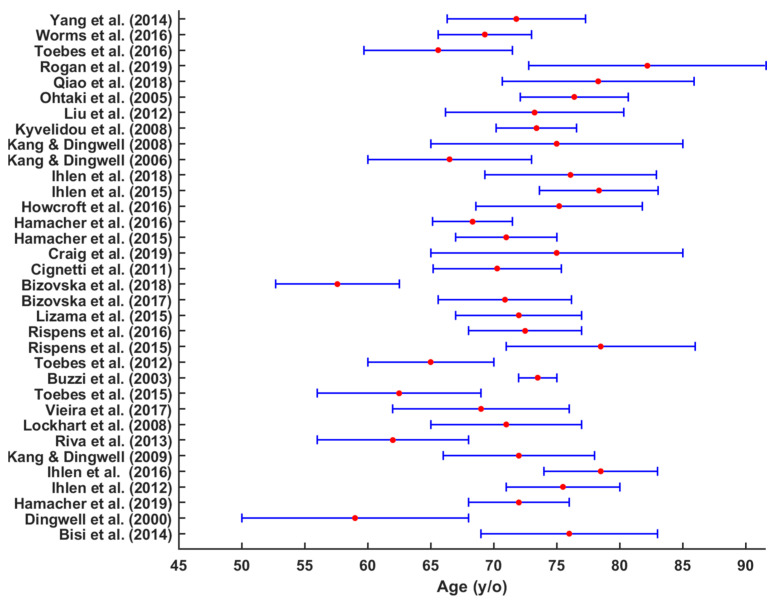
Mean and SD age (years) of older participants in the studies.

Thirty papers reported the gender of participant groups. Terrier and Reynard [43] and Reynard and Terrier [54] had an equal number of both male and female participants, while Bizovska, Svoboda [9] and Kyvelidou, Kurz [47] included only female participants (Table 1). Generally, in most of the studies, female participants were more prevalent than male [3,7,9,31,34,35,36,38,39,42,45,51,52,53,56,59,61]. The average age range for older participants were 70.6 ± 6 (65–76.2) years old (Figure 2) and 25 ± 5 (20–30) years old for younger participants [3,6,7,27,28,29,31,33,40,41,42,43,48,52,53,54,55,56,57,58,62].

#### 3.1.2. Sample Size

The sample sizes ranged from 12 [50] to 139 [32] participants. In two studies, the number of participants in each group was very low, for example, 5 or less in each group [33,50]. In 10 studies, the number of participants in total was more than 100 [3,28,30,31,32,34,35,42,43,54,55].

As shown in Table 1, the F-NF comparison was considered in 10 studies [27,28,29,30,31,32,33,34,35,36,37,38,39], and the YO comparison was conducted in 14 studies [3,6,7,9,38,40,41,42,43,44,45,46,47,48,49]. Six studies included F-NF and YO comparisons [33,50,51,52,53,54].

#### 3.1.3. Fall Risk Assessment Tools

The eligibility criteria used to define both healthy and fall-prone older people varied substantially across studies. Fall risk was assessed in a variety of ways, including the history of falling and clinical fall risk assessments/questionnaires (Table 2). Faller definitions in studies were classified into six categories: at least one fall history in 3 months prior to the measurement [28], at least one fall within 6 months [32,33,34], more than two fall incidents within six months prior to the study but were uninjured at the time of the experiment [50], at least one fall history within the last 12 months [7,32,33,40] and at least two falls during the previous year [31,38,42]. Additionally, there were two studies with no falls in the past 12 months [42,55]. Nine studies did not provide a coherent definition of fallers [6,7,41,42,43,56,57,63,64].

Fall risk questionnaires included: anamnestic questionnaire focusing on participants’ physical condition [28], fall history questionnaire [3,28,29,30,31,33,38,40,42,50], self-reported ability to walk one mile at any pace with minimum rest, the Survey of Activities and Fear of Falling in the Elderly (SAFE) [3,63], the Movement Specific Reinvestment Scale (MSRS) [38], the Falls Efficacy Scale International (FES-I) [38], depression (CES-D) [3], the Longitudinal Aging Study Amsterdam Physical Activity Questionnaire (LAPAQ) [30] and mini mental state examination score (MMSE) [37,38,56,57,58,65].

Objective clinical balance assessments were mostly used within inclusion and exclusion criteria and included the Tinetti Balance Assessment Tool (TBAT) [28,32], single leg stance test, Timed Up and Go, 10 m Walk Test, Figure 8 Walk, Four Square Step [63], physical activity (Freiburger Fragebogen zur körperlichen Aktivität), proprioception (joint position sense), peripheral sensation (mechanical and vibration detection threshold), balance performance (static balance on force plate) and muscular fitness (instrumented sit-to-stand test), test of cognition performance (Stroop test), health status (SF12) and pain status (painDETECT, SES) [3] (Table 2).

#### 3.1.4. Treadmill (TM) versus Overground (OG) for Estimating Non-Linear Dynamics

**Figure 3:**
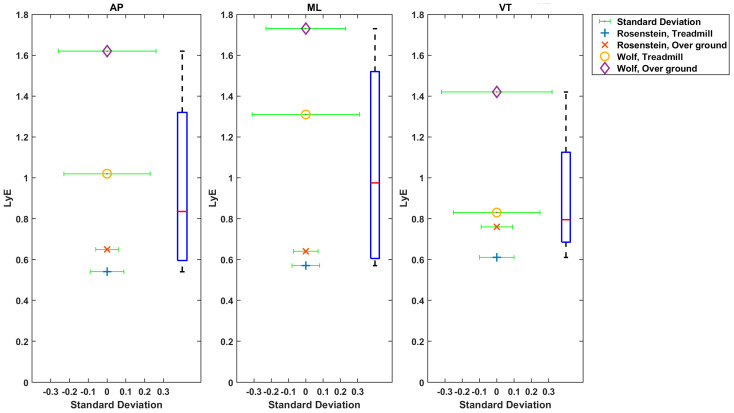
Estimated Mean (x axis) and SD (y axis) LyE values by Rispens et al. (2016).

In 24 studies, participants walked on a TM [3,7,17,30,31,33,38,40,41,42,43,44,45,46,47,48,50,51,52,53,54,55,58,60,61,66], while 13 studies examined OG walking [6,9,29,32,34,35,36,37,39,49,56,57,59,63]. OG and TM walking were compared in three studies, where LyE values were higher for OG compared to TM walking using both Rosenstein’s and Wolf’s methods (Figure 3) [6,29,59].

### 3.2. Data Collection Modality and Kinematic Variables Analysed

Data collection modalities were divided into four categories: Inertial sensors or accelerometers; goniometers; force plates; and motion capture using passive marker-based systems (Vicon, Qualisys) and video. In 25 out of 44 studies, inertial sensors or accelerometers were used. In these studies, the sensor was placed on: the lower back, at L5 level [3,38,50,61]; on the lower back, at L5 level and on both shanks, approximately 15 cm above the malleolus [28,32]; on the dominant forefoot and upper thoracic spine [3]; near the right anterior superior iliac spine (ASIS) [33]; to the back, just below the shoulder [29,32,33,57]; with an elastic belt around the waist and set along the lumbar spine [31,37,56,58,59]; at sacrum with an elastic band [58]; over the posterior surface of the lumbar spine at approximately the level of L5 and on the lateral surface of the distal shank, superior to the ankle joint [51], forefeet and trunk [45], the posterior head with a band, posterior pelvis with a belt, and lateral shank, just above the ankle, with a band [34]; right ankle [52]; and at the fourth spinal process of the lumbar spine [37].

From these inertial sensor studies, variation in the derived variables used to make the nonlinear dynamic calculations existed. Nine different categories of data were analysed from inertial sensors across these studies:

VT, AP, and ML trunk accelerations [3,29,30,34,36,45,50,56,58];

3D angular velocity [7,31,57];

Trunk accelerations in the AP and ML directions [30,51];

Trunk VT and ML accelerations and 3D angular velocity [31,57];

Trunk acceleration in ML [58];

AP accelerometer signal close to the hip [33];

AP, VT and ML trunk linear acceleration and angular velocity [36,45];

AP ankle acceleration [52];

VT and ML trunk acceleration [37].

Goniometers were used in a single study [27] and placed to measure sagittal plane movements of knee, ankle and hip joints.

Fifteen studies used motion capture with passive marker based systems [38,39,40,41,42,44,46,49,50,53,60,61,63] or video [7,47]. Studies analysed different variables included: T10 3D velocity [63]; 3D position and velocity of the centre of mass including the centre of pressure (by using pressure plate) [50]; 3D linear and angular velocities of trunk, pelvis, thigh, shank and foot segments [41]; VT acceleration, AP and ML trunk velocities [42]; VT displacement of hip, knee, ankle and relative knee angles [7]; 3D angular position of hip and ankle [44]; 3D linear and angular velocity and acceleration of trunk [60]; ML angular velocity of the thigh, shank and foot [47]; 3D C7 vertebrae marker velocity time series [53]; AP ankle, knee and hip angle [49]; ML trunk velocity and 3D trunk kinematic [61]; Centre of mass velocity signal [38]; ML, VT and AP trunk positions and 3D rotational movement of the trunk [39].

### 3.3. Nonlinear Dynamic Analysis

Studies that performed an assessment of fallers versus non-fallers reported seven methods of nonlinear dynamic analysis which are Maximum Lyapunov (LyE, short term Lyapunov and long term LyE), Floquet multipliers (FMs), recurrence quantification analysis (RQA), correlation dimension (CD) [7], multiscale entropy (MSE) [3,6,32,34], sample entropy (SEn) [42] and Shannon entropy (ShE) [32]. Among all the studies, LyE has been mostly used [1,2,4,7,9,17,26,28,37,38,40,42,43,45,46,47,48,49,51,52,55,56,57,60,61,62,64,65,66,67].

Twenty-one studies used Rosenstein’s method [3,6,9,27,28,31,33,34,37,38,39,40,41,42,43,46,49,50,52,53,54,55,60,61,63]. One study used Kant’s method [48]. Two studies used Rosenstein’s and Kantz’s methods together [40,44]. One study used Rosenstein, Kant and Ihlen methods [29]. In three studies Wolf’s method was used [4,38,58]. In one study, Rosenstein’s and Wolf’s methods were used [57]. Rispens, Van Dieën [57] showed different values of LyE estimation using Rosenstein’s method and Wolf’s method (Figure 3). In some studies, short term LyE was estimated [3,7,36,40,57,65] or both short and long terms were estimated [6,30,33,41,43,55,56,62]. Finally, max LyE or finite time LyE were estimated [7,27,33,37,42,43,47,48,52,56,57,58,61].

Hurmuzlu’s method [67] was used to calculate FMs across the studies [37,41,43,52].

MSE was calculated from Ihlen’s method [35] and Richman’s method [30,68].

### 3.4. Nonlinear Dynamic Variable Values

All studies reported lower local dynamic stability in older adults than younger adults, where greater sensitivity to local perturbations could be found in larger LyE values [3,6,7,9,12,27,28,29,31,33,34,37,38,39,40,41,42,43,47,48,52,53,54,55,56,57,59,60,61,63]; thus, they concluded that dynamic stability is lower in older people than younger adults. There are no references for acceptable ranges of LyE due to the different methods of calculation used. In all the studies, the lowest value of LyE meant more dynamic stability. In most studies reported, LyE values were positive. However, in Ihlen, Sletvold [40], negative and positive values were reported.

**Figure 4:**
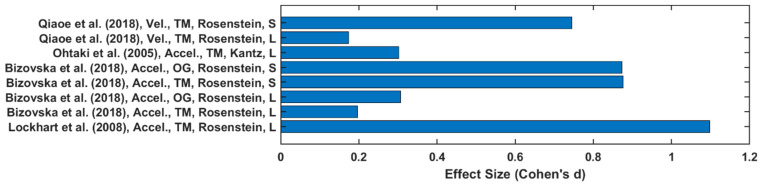
Effect size of LyE between younger and older groups.

**Figure 5:**
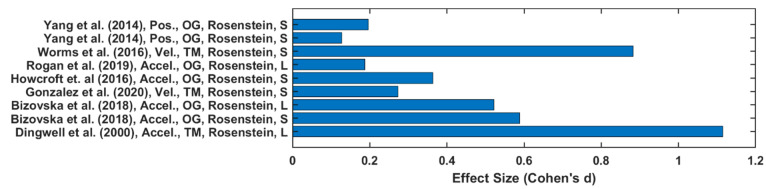
Effect size of LyE between healthy and fall-prone older adults’ groups.

Twenty-one out of forty-five studies reported LyE value of trunk acceleration or velocity [3,9,27,28,29,30,31,33,37,38,41,42,43,47,53,54,55,56,57,59,60,61,63]. In papers reporting a YO comparison, the estimation of LyE values calculated from acceleration data produced higher effect size values on average (0.61 ± 0.39) than velocity (0.46 ± 0.4), as depicted in Figure 4. Additionally, the OG walking effect size (0.59 ± 0.4) is higher than TM walking (0.56 ± 0.39). However, in F-NF studies, LyE calculated from velocity data has been shown to produce a higher effect size value (0.58 ± 0.43) than from acceleration data (0.55 ± 0.35), as shown in Figure 5. TM walking effect size (0.76 ± 0.43) is higher than OG walking (0.37 ± 0.19).

**Figure 6:**
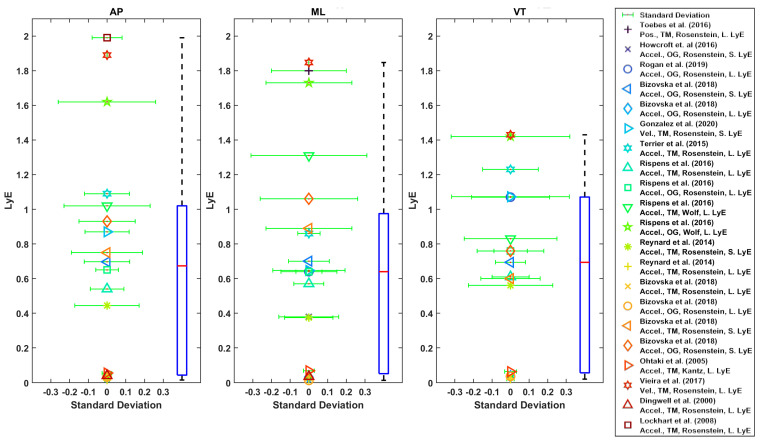
Reported Trunk Short-Term LyE or Long-Term LyE for healthy older adults.

**Figure 7:**
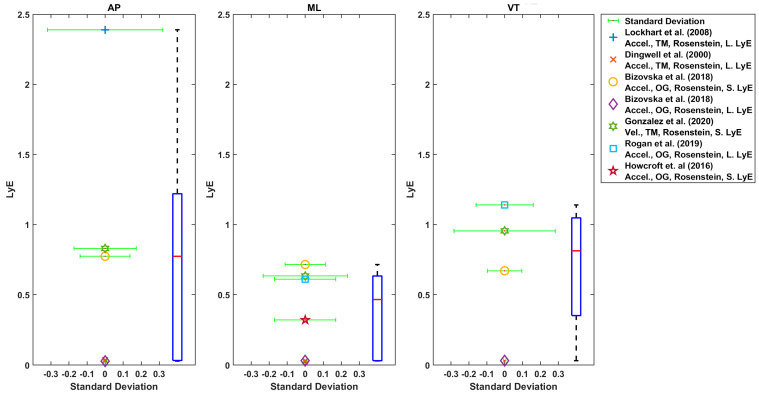
Reported Trunk Short-Term LyE or Long-Term LyE for fall-prone adults.

**Figure 8:**
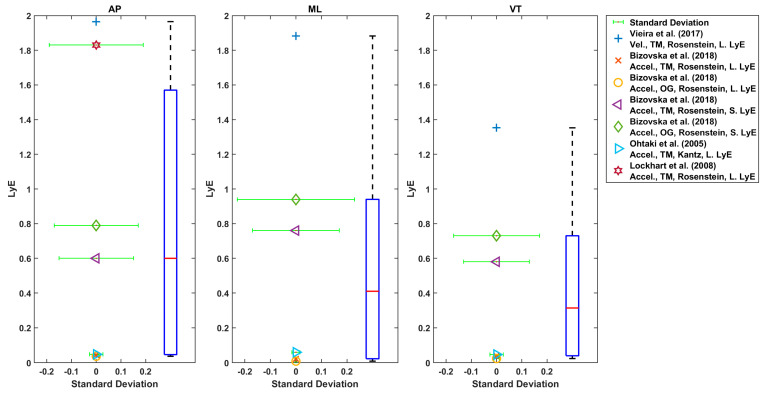
Reported Trunk Short-Term LyE or Long-Term LyE for younger adults.

**Figure 9:**
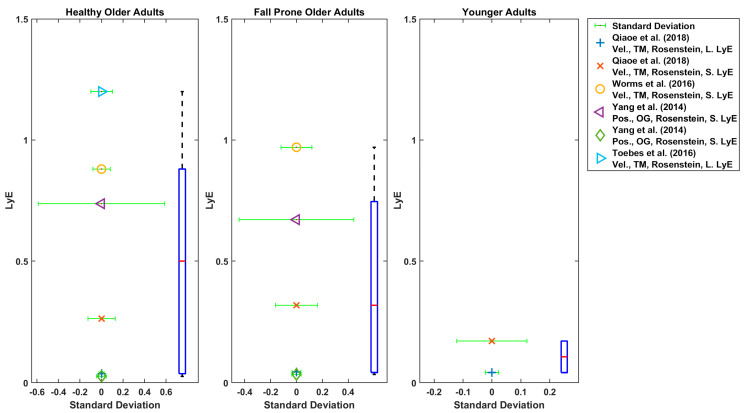
Reported Trunk Short-Term LyE or Long-Term LyE for healthy older, fall-prone and younger adults.

Generally, AP and ML directions were used for LyE estimation in these studies (Figure 6, Figure 7, Figure 8 and Figure 9) [27,28,29,30,42,43,56,57,61,63]. In one study, only AP direction was used [33]. In some studies, all three directions were used [9,28,29,42,43,56,57,59,63].

The boxplot of LyE values for healthy older adults estimated using Rosenstein’s or Wolf’s method is shown in Figure 6. Some studies used acceleration, and some used velocity of the trunk. The median values for LyE were 0.67 (18 values), 0.64 (20 values) and 0.69 (17 values) for AP, ML and VT directions, respectively (Figure 7).

Accordingly, the median of the LyE for fall-prone older adults using Rosenstein’s method was 0.77 (5 values), 0.61 (6 values) and 0.81 (4 values) for AP, ML and VT directions, respectively.

Considering the LyE for younger adults using Rosenstein’s or Kantz’s method, the median value was 0.6 (7 values), 0.41 (6 values) and 0.31 (6 values) for AP, ML and VT directions, respectively (Figure 8). Furthermore, using Rosenstein’s method, the median of estimated LyE from 3D velocity was 0.1 (2 values), 0.5 (5 values) and 0.32 (values) for younger adults, older healthy adults and fall-prone older adults, respectively (Figure 9).

As shown in Figure 6, Figure 7, Figure 8 and Figure 9, the reported values from across all reviewed studies do not clearly distinguish between the LyE values of F-NF and YO, despite each study showing LyE higher in fallers compared to non-fallers and older versus younger adults.

Nonlinear dynamic measures that have been used less often in studying falls in older populations are FMs, CD, RQA, MSE, ShE and SEn. Kang and Dingwell [41,60] and Granata and Lockhart [50] reported higher values of FMs for older adults compared to younger adults, which indicated more dynamic stability in younger adults rather than older adults. In Kang and Dingwell [41], FMs were larger (lower dynamic stability) in the superior segments compared to inferior segments and in older adults compared to younger adults. Bisi, Riva [6] reported that FMs did not have significant differences between older and younger populations. Two studies reported that FMs had negative predictive validity in their fall prediction model [12,39]. Granata and Lockhart [50] used FMs and reported that the maximus FM was a good indicator of fall-prone older adults; however, it could not capture the effect of speed on stability. In contrast, Kang and Dingwell [60] showed that FMs decreased at slower speeds and increased at faster speeds in both younger and older populations. In some studies, both LyE and FMs were used [13,41,43,62] and reported larger LyE and FMs in older adults.

In different studies, different combinations of nonlinear dynamic measures were used; however, IC, ShE and MSE were consistently lower in faller compared to non-faller groups [32,35]. Bizovska, Svoboda [32] used ShE, MSE and IC and reported only ShE could distinguish faller from non-faller older adults. Vieira, Rodrigues [42] used both LyE and SEn in studying healthy older adults. Riva, Toebes [30] used MSE and RQA and proposed MSE and RQA to be positively associated with the fall history. Bisi, Riva [6] used LyE, FMs, RQA and MSE and suggested that RQA better-distinguished gait dynamic stability in stable healthy adults. Buzzi, Stergiou [7] reported higher LyE and CD values in older populations. Bizovska, Svoboda [32] estimated MSE, IC and ShE and reported ShE seems to be sufficient in fall risk prediction. Bizovska, Svoboda [9] considered MSE and LyE and stated significant age-related differences in gait were found for LyE.

## 4. Discussion

The aim was to summarise the variety, characteristics and range of nonlinear dynamic measurements used to distinguish the gait kinematics of healthy older adults and older adults at risk of falling. There is a need to improve existing methods of estimating gait stability to improve the effectiveness of detecting fall risk and predicting falls. This review has helped to ascertain the variety and range of nonlinear dynamic measurements used to characterise gait for healthy older people and older people at risk of falling.

Among all nonlinear dynamic analyses used, LyE was the most common. All studies reported higher LyE and CD for older adults versus younger adults and fall-prone older adults versus non-faller older adults. Hence, these measures should be considered robust. However, values between studies cannot be compared since the methodology used, kinematic characteristics analysed and data analysis procedures differed greatly. Three papers considering FMs reported higher values for older adults compared to younger adults, which indicated more dynamic stability in younger adults [41,50,60]; however, no work with fallers and non-fallers has been published to date. In two studies, entropy and index of complexity reported lower values in older people with a history of falls [32,35].

In the literature, nonlinear dynamic measures have been calculated for a variety of kinematic variables such as marker position, joint angular displacement and velocity, the centre of mass and acceleration. In agreement with the motor control theory proposed by Bernsteĭn [69], the dynamics of movements are not only dependent on the task-related constraints (e.g., walking speed) but also on the biomechanical constraints of the investigated structure (e.g., kinematic variables) [70,71]. However, these kinematic variables (e.g., knee, hip, ankle angle, velocity, etc.) do not demonstrate the same behaviour toward changes in speed and conditions. There is still a lack of clarity as to which kinematic variables are more sensitive for distinguishing fallers from non-fallers. A concern when using nonlinear dynamic analysis for experimental data is the length of the time series analysed [25,48,72]. Different studies used various data lengths. Other concerns pertain to parameters such as time series filtration, normalisation, and input variables like embedding dimension (ED) and time delay (TD). Each of these parameters has a direct effect on LyE estimation [22,23], for example. Different studies used various data lengths, ED and TD. Some of them filtered and/ or normalised time series, and others did not, and this is the same for data normalisation. This, in turn, makes it challenging to compare values across studies.

### 4.1. Study Design Characteristics

#### 4.1.1. Sample Size and Characteristics

Since women are at higher risk of falls compared to men [1], studies should consider examining sex differences in more detail. To date, no studies examined male versus female differences.

We did not specify a priori an age cut point as an inclusion criterion for papers; rather, we used the interpretation of age in the primary studies included in the review. The age range of younger adults (20–30) and particularly older adults (mean of studies: 65–76.2; age range across all studies: 50–90) varied significantly among the studies. Varied age ranges for those classified as ‘older’ may be related to the large range of nonlinear dynamic values reported in the literature. In addition, the number of participants selected in each group differed. The number of participants in each group should be equal to avoid any possible bias. There are standard methods available for the calculation of sample sizes to estimate expected differences between groups, and these should be adopted for all studies [73].

#### 4.1.2. Fall Risk Assessment Tools

All studies were cross section in nature; therefore, the method of determining fallers from non-fallers is key to comparing the consistency of findings between different research groups. In this review, there was no consistently used fall risk assessment tool to identify the ‘fall groups’, and thus, there is an inconsistent classification of fall risk across studies, which, if nonlinear dynamic measures are associated with fall risk, makes it harder to compare estimates across studies. Consistent and valid fall risk assessment tools need to be studied in future works to standardise across studies, and we suggest using the ProFANE definition of falls and methods for ascertaining falls [73].

#### 4.1.3. Treadmill versus Overground

Biomechanical parameters and nonlinear dynamic measures obtained from TM walking are known to differ from those gained from OG walking [8,9,27,57,74]. Therefore, it is not possible to compare values obtained from TM and OG walking studies.

Based on both practicality and the data required to perform nonlinear dynamic measures, TM walking facilitates controlled experimental conditions while being able to distinguish less stable gait patterns. For older people in the studies reviewed, dynamic stability was increased by small but statistically significant amounts when walking on the TM compared to OG due to artificially stabilised natural locomotor kinematics [8,9,27,57,74]. In most of the studies, TM walking is used because a large amount of continuous data is needed for the calculation of all nonlinear dynamic measures and especially for LyE [23,27,67]. TM studies still found increased LyE for older versus younger adults. Therefore, while reducing values, they can still distinguish the groups; however, the relative effect of TM walking should be considered when using motorised treadmills and, more importantly, in fall prevention intervention strategies.

### 4.2. Data Collection Modality and Kinematic Variables Analysed

Until recently, LyE and FMs have been calculated on data collected in a movement laboratory with motion capture systems, making them relatively cost and time expensive to evaluate [17]. With the availability of inertial sensors, it has been cheaper to conduct these kinds of studies. The effect size of LyE values estimated from acceleration or velocity during OG walking was higher in the YO and F-NF studies, which suggests that more significant differences between YO and F-NF could be captured while participants were walking on the ground compared to on a treadmill. While the evidence is compelling, it is still not clear if different kinematic variables such as acceleration, velocity or position reveal the same properties of the nonlinear characteristics of the gait, and this is an area for future research. Thus, TM and OG studies should continue in parallel.

Inertial sensors were most commonly used (compared to camera-based methods) to study falling in older adults because they have the ability to collect data outside the laboratory environment [75,76]. Different makes of inertial sensors such as Dynaport Hybrid [19,30,32], OPALS [6], Delsys Inc [28,32] and Xsens [3,45,52,59] were used across studies. In addition, a varying number of sensors were used. In some studies, only one inertial sensor (placed on the trunk) was used; in others, two or three inertial sensors were used (placed on the trunk and ankles). Using different sensors with various specifications (sensors’ range and sample rate differences) and different sensor placements on the body can be another reason for the variability in the reported NDA measures values [77]. An inertial sensor should be placed so that the maximum movements and signals can be captured [75,76]. Overall, the possibility to use inertial sensors to determine nonlinear characteristics of gait is a promising field. However, the methodology for data collection, processing and analysis should be standardised to facilitate better comparisons between studies and the generation of reference values for the field.

Inconsistency in data collection modality and process hinders the ability to compare nonlinear dynamic measures across studies and identify ‘normal’ values. In addition, it is still questionable what kinematic variables (position, velocity and acceleration), collection modalities and sensors’ positions result in the most sensitive and specific nonlinear dynamic measures of walking related to ageing and falling. Future work might explore this area in a systematic way and try to standardise the acceleration’s position in this kind of study.

### 4.3. Nonlinear Dynamic Analysis

Among all the aforementioned nonlinear dynamic measures, LyE using Rosenstein’s method has been most commonly used to investigate gait characteristics in older populations. However, Cignetti, Decker [44] reported that Rosenstein’s method provided less sensitivity than Wolf’s method to capture age-related differences from small gait data set. They concluded that Wolf’s method is more appropriate for the estimated LyE from small gait data sets [44]. Hence, there is still information missing on which method is more sensitive to differences between fallers and non-fallers.

There are not yet any population averages, nor are there reference values created under an agreed laboratory protocol that would allow a fixed and robust interpretation of the nonlinear dynamic measures due to different study designs and methods of calculation being used in the literature.

Although it is vital to reconstructing phase space with optimum TD and ED for LyE estimation [25], there are no mathematical rules for selecting the ‘correct’ values for TD and ED to reconstruct the phase space; rather, some recommendations have been suggested for their estimation [78]. For the majority of literature published in this area, authors make reference to the algorithms for estimating ED and TD and Theiler window but rarely report the actual values when reconstructing and subsequently quantifying characteristics of the attractor state. Mathematically, it is presented that decreasing the ED lead to self-intersections of the reconstructed trajectory [78]. Therefore, this can be an area of future work when or if attempting to standardise LyE calculation.

There was consistency in calculations methods of other nonlinear dynamic measures utilised were MSE, RQA, IC, and CD [3,4,32,34]. However, choosing optimum input parameters for each of them has a huge effect on each of their values and still is a challenge.

### 4.4. Nonlinear Dynamic Variable Values

While LyE has been most widely used, other nonlinear dynamic measures of gait have the potential to distinguish fallers from non-fallers. The relative sensitivity and specificity of these measures are yet to be fully determined and will be an avenue for future research.

Although all studies reported higher LyE for an older population, there was not a consistence range for their values. This is likely because of using different LyE estimation methods and/or different data acquisition, and/or even using different kinematic variables as reported above. In some studies, using velocity and, in others, using acceleration showed more sensitivity among groups. Reported LyE values in YO comparison studies, indicating that acceleration and OG walking modality in nonlinear dynamic measures for gait are more sensitive to the changes due to ageing. However, reported LyE values in F-NF studies suggest that using velocity while participants were walking on a treadmill could better distinguish fallers from non-fallers. There is a possibility that these inconsistencies in the estimation of LyE for gait in YO and the elderly came from the different methodological approaches adopted in the studies. Therefore, this led to high variability in reported LyE values for younger and older adults. Future work needs to standardise the LyE estimation method.

In contrast to LyE, there were just a few studies using other nonlinear dynamic measures. Among them, the inconsistency in the results across studies suggests that FMs were a less sensitive measure for studying changes in the gait of older adults. In addition, one study stated that CD captured a significant difference between younger and older adults’ gait [7]. However, the strength of evidence is lacking due to the limited number of studies and varied methods.

MSE was commonly used for the quantification of complexity, among other three entropy analyses applied across studies [3,6,32,34]. Some studies reported that MSE and measures of RQA have been associated with fall history [30] and that MSE and ShE can be used as fall risk predictors [32,35]. However, same as with the LyE values, there are not any normative entropy values with which to compare values among studies. Entropy algorithms are sensitive to their input parameters of tolerance window r, vector length m, time series length N and number of scales [20]. Inconsistency in these input parameters between studies has led to conflicting results between studies. Hence, further studies with consistent data processing methodologies need to be carried out in this area.

Variations in the methodology and data analysis procedures cannot support a standard nonlinear dynamic measures range for populations, which can be used in clinical settings to predict fall risk. Currently, we have evidence that preferred speed is better prediction than fixed speed in the data collection [27], but the relevance of other components of test protocols is not known. We cannot be sure what protocol should be used for generating reference values. For example, OG/ TM, accelerometer/ velocity/ position, nonlinear dynamic measures (LyE/ FMs/ RQA/ MSE) and their estimation’s methods. More research is needed to determine which protocols are most sensitive and specific for the prediction of falls.

### 4.5. Limitations

Our review had a number of limitations and constraints. Only one reviewer appraised the quality of papers, and because of the breadth of research methods being reported in the papers, quality of assessment of methods was challenging, and it was not possible to use standard techniques to assess quality, pool results and estimate heterogeneity. We used the items in the PRISMA reporting guideline to provide a framework for quality assessment but recognise that ideally we should have used more detailed quality assessment instruments. We included two databases only, but these are both considered to have broad coverage of the literature. Nevertheless, we believe the review scope and results reflect progress in the field well.

## 5. Conclusions

Although NDA for determining specific parameters of mobility can assess function and stability in the elderly, measures have hardly been taken up in clinical settings because of their unclear sensitivity and specificity, together with the time and effort required for their use. There is much variation in reported nonlinear dynamic values across studies that can be attributed to three main factors: (i) experiment design, (ii) fall risk assessment tools and (iii) variables analysed and nonlinear dynamic measures estimation method. Due to the robustness of findings, in future studies, we suggest that it is worth standardising data collection, variable definition and the estimation methods of nonlinear dynamic measures. This will lead the field towards better acknowledging the possibility of clinically relevant nonlinear dynamic values for identifying fall risk.

## Figures and Tables

**Table 1:** Equal gender/ Sex ratio, sample size, participant’s gender (Female, Male or not mentioned (nm)), overground (OG) or on the Treadmill (TM) or Both (Both) cohorts in studies in corporation F-NF or YO comparisons.

Author (Year) [Reference]	F-NF	YO	Equal Gender/Sex Ratio	Sample Size	Female/Male	TM/OG/Both
Bisi et al. (2014) [6]	no	yes	no	30	nm	TM
Dingwell et al. (2000) [27]	yes	no	no	24	7/17	Both
Bizovska et al. (2018) [28]	yes	no	no	139	nm	OG
Gonzalez et al. (2020) [63]	no	no	no	34	nm	TM
Granata et al. (2008) [50]	yes	yes	no	12	nm	TM
Hamacher et al. (2019) [3]	no	yes	no	102	52/50	OG
Ihlen et al. (2012) [40]	no	yes	no	20	8/12	TM
Ihlen et al. (2016) [29]	yes	no	no	71	nm	OG
Kang & Dingwell (2009) [41]	no	yes	no	25	11/14	TM
Lockhart et al. (2008) [33]	yes	yes	no	13	nm	TM
Riva et al. (2013) [19]	yes	no	no	131	nm	TM
Vieira et al. (2017) [42]	no	yes	no	87	46/41	TM
Toebes et al. (2015) [31]	no	no	no	134	85/49	TM
Buzzi et al. (2003) [7]	no	yes	no	20	20/0	TM
Terrier et al. (2015) [43]	no	yes	yes	100	50/50	TM
Toebes et al. (2012) [55]	no	no	no	134	85/49	TM
Rispens et al. (2015) [56]	no	no	no	110	77/33	OG
Rispens et al. (2016) [57]	no	no	no	18	7/11	Both
Lizama et al. (2015) [58]	no	no	no	19	7/12	TM
Bizovska et al. (2017) [32]	yes	no	no	139	nm	OG
Bizovska et al. (2018) [9]	no	yes	yes	139	nm	Both
Cignetti et al. (2011) [44]	no	yes	no	14	5/9	TM
Craig et al. (2019) [51]	yes	yes	no	65	48/17	TM
Hamacher et al. (2015) [45]	no	yes	no	39	26/13	OG
Hamacher et al. (2016) [59]	no	no	no	32	21/11	OG
Howcroft et al. (2016) [34]	yes	no	no	100	56/44	OG
Ihlen et al. (2015) [36]	yes	no	no	71	nm	OG
Ihlen et al. (2018) [35]	yes	no	no	319	162/157	OG
Kang & Dingwell (2006) [46]	no	yes	no	20	nm	TM
Kang & Dingwell (2008) [60]	no	no	no	36	12/24	TM
Kyvelidou et al. (2008) [47]	no	yes	yes	20	20/0	TM
Liu et al. (2012) [52]	yes	yes	no	12	7/5	TM
Ohtaki et al. (2005) [48]	no	yes	no	59	26/33	OG
Qiao et al. (2018) [53]	yes	yes	no	33	19/14	TM
Reynard et al. (2014) [54]	yes	yes	yes	100	50/50	TM
Rogan et al. (2019) [37]	yes	no	no	26	nm	OG
Segal et al. (2008) [49]	no	yes	no	19	5/14	TM
Toebes et al. (2016) [61]	no	no	no	16	9/7	TM
Worms et al. (2016) [38]	yes	no	no	28	20/8	TM
Yang et al. (2014) [39]	yes	no	no	187	187/0	TM

**Table 2:** Fall risk assessment tools used across studies.

Questionnaire	Description	Clinical Assessment	Description
Anamnestic questionnaire [28]	Focusing on participants’ physical condition and fall history in the 3 months prior the measurement.	Tinetti Balance Assessment Tool (TBAT) [28,32]	Assesses the gait and balance in older adults and perception of balance and stability during activities of daily living and fear of falling.
Fall history questionnaire [3,28,29,30,31,33,38,40,42,50]	Self-reported medical questionnaires also indicated participants had recent histories of falling (at least one fall within 6 months, 3 months or one year).	Single leg stance test [63]	Assesses static postural and balance control
Survey of Activities and Fear of Falling in the Elderly (SAFE) [63]	Includes one scale and one subscale: the scale asks participants whether they perform a series of 11 activities of daily living and, if so, their level of fear of falling during the activity (Fear of Falling subscale). A separate subscale (Activity Restriction subscale) asks participants to rate the extent to which they currently engage in each activity relative to five years ago.	Timed Up and Go [63]	Determines fall risk and measures the progress of balance, sitting to standing and walking
The Movement Specific Reinvestment Scale (MSRS) [38]	It is a measure of the propensity for movement-related self-consciousness and for conscious processing of movement and was used to try to discriminate elder fallers from non-fallers.	10 m Walk Test [63]	It is a performance measure used to assess walking speed in meters per second over a short distance. It can be employed to determine functional mobility, gait and vestibular function.
The Falls Efficacy Scale International (FES-I) [38]	A measure quantifying an individual’s concern about falling during various tasks, yielding a score between 16 (low concern about falling) and 64 (high concern about falling).	“Figure 8” Walk [63]	Measures the everyday walking ability of older adults with mobility disabilities. It tests a participant’s gait in both straight and curved paths.
The Longitudinal Aging Study Amsterdam Physical Activity Questionnaire (LAPAQ) [30]	A 31-point questionnaire that covers the frequency and duration of walking outside, bicycling, gardening, light household activities, heavy household activities, and a maximum of two sport activities during the previous two weeks.	Four Square Step [63]	Assesses dynamic stability and the ability of the subject to step over low objects forward, sideways, and backward.
Mini mental estate examination score (MMSE) [37,38,56,57,58]	A 30-point questionnaire that is used extensively in clinical and research settings to measure cognitive impairment.	Clinical balance assessment (static balance on force plate) [3]	Determine a patient’s ability (or inability) to safely balance during a series of predetermined tasks. It does not include the assessment of gait.

## Data Availability

Not applicable.

## Abbreviations

LyE	Lyapunov Exponent
FMs	Fouquet Multipliers
MSE	Multiscale Entropy
RQA	Recurrence quantification analysis
TM	treadmill
OG	overground
AP	anterior-posterior
VT	vertical
ML	medio-lateral
YO	younger adults and older adults
F-NF	Fall-prone older adults and non-faller older adults
SAFE	the Survey of Activities and Fear of Falling in the Elderly
MSRS	Movement Specific Reinvestment Scale
FES-I	The Falls Efficacy Scale International
CES-D	depression
LAPAQ	The Longitudinal Aging Study Amsterdam Physical Activity Questionnaire
MMSE	Mini mental state examination score
TBAT	Tinetti Balance Assessment Tool
SD	Standard deviation
Sen	Sample Entropy
CD	Correlation Dimension
ShE	Shannon Entropy
